# Triple Negative Breast Cancer: An Analysis of the Subtypes and the Effects of Menopausal Status on Invasive Breast Cancer

**DOI:** 10.3390/jcm11092331

**Published:** 2022-04-22

**Authors:** Reiki Nishimura, Tomofumi Osako, Yasuhiro Okumura, Masahiro Nakano, Hiroko Otsuka, Mamiko Fujisue, Nobuyuki Arima

**Affiliations:** 1 Department of Breast Oncology, Kumamoto Shinto General Hospital, Kumamoto 862-8655, Japan; tohsako630108@yahoo.co.jp (T.O.); sigeoku@yahoo.co.jp (Y.O.); mnakano1020@gmail.com (M.N.); hirokotuka.o@gmail.com (H.O.); mamiko1014poo@yahoo.co.jp (M.F.); 2 Department of Pathology, Kumamoto Shinto General Hospital, Kumamoto 862-8655, Japan; nobuari0816@yahoo.co.jp

**Keywords:** breast cancer, triple negative, menopausal status, Ki-67, disease-free survival

## Abstract

Background: Triple negative breast cancer (TNBC) is a subtype of breast cancer which lacks hormone receptor (HR) expression and HER2 gene amplification and is the most aggressive subtype, with a heterogeneous genetic profile. The aim of this retrospective study was to evaluate the clinical significance of menopausal status in breast cancer cases with TNBC. Methods: Primary breast cancer patients who underwent curative surgery were enrolled in this retrospective study. A total of 5153 invasive breast cancer cases with Stage I–III were analyzed. The distribution of cases according to the menopausal status and subtypes was investigated and the clinicopathological characteristics and prognosis were compared between pre- and postmenopausal TNBC patients. Results: TNBC was frequently seen in postmenopausal patients and Luminal B and Luminal/HER2 subtypes were more common in premenopausal patients. There was no difference in DFS in the Luminal A/B and HER2 subtypes, but a significant difference was seen in the TNBC patients. Premenopausal patients with TNBC frequently had an overexpression of the p53 protein, a significantly higher Ki-67 index value, and a higher nuclear grade. A multivariate analysis revealed that menopausal status, nodal status, and tumor size were significant factors for DFS in TNBC cases. Conclusion: Menopausal status significantly correlates with breast cancer subtypes. TNBC was often seen in postmenopausal patients and these patients tend to have more favorable factors and a better DFS than premenopausal patients. These findings suggest that menopausal status is an important factor for evaluating biology and prognosis in TNBC cases.

## 1. Introduction

Recent statistical findings show that triple negative breast cancer (TNBC) accounts for about 10–20% of all breast cancer cases. TNBC tests negative for estrogen receptor (ER), progesterone receptor (PgR), and does not have an excess level of the HER2 protein. Moreover, these cases are aggressive in nature, tend to have an unfavorable prognosis, and have shorter survival compared to the non-TNBC cases [1,2]. Since the tumor cells lack the receptors ER, PgR and HER2, common treatments such as endocrine therapy and anti-HER2 therapy are ineffective. New drugs have been developed to treat TNBC that enhance the immune system (PD-1 and PD-L1) and PARP. However, chemotherapy is still the main component in systemic adjuvant therapy for all primary breast cancer cases.

TNBC is commonly seen in women 50 years of age and younger and the other breast cancer subtypes tend to occur more often in women aged 60 or older. One study reported [3] that there was no correlation between age and invasive disease-free survival (DFS) or overall survival (OS) after adjuvant chemotherapy. However, a different study [4] reported that shorter survival times, distant DFS, and breast cancer-specific survival were found in the older age group with TNBC. A multivariate cox regression analysis was conducted to compare cases who received chemotherapy with cases that did not receive chemotherapy, and found that there was a significant improvement in OS in the cases that received chemotherapy [5]. As for patients with lymph node involvement after NAC, the premenopausal status in grade III tumors correlated with a poor prognosis [6]. Carlson et al. [7] reported that there was a worse outcome for premenopausal BRCA mutation carriers compared with the postmenopausal BRCA mutation carriers. Freedman et al. [8] reported that older patients had unfavorable breast cancer outcomes, regardless of the disease subtype (including TNBC) and stage. Moreover, a comprehensive review of the data revealed that there is no clinical consensus on menopausal status and prognosis in TNBC.

Breast cancer in younger women can be more aggressive than in older women, and the menopausal status has limited predictive power for distant recurrence and OS in HR positive cases [9]. The number of postmenopausal breast cancer cases is increasing in Japan; therefore, knowing how menopausal status affects TNBC is important for future treatment. Moreover, an understanding of the heterogeneity within the TNBC subtype is important for managing the disease. In this retrospective study the clinical significance of menopausal status was evaluated in breast cancer cases with TNBC.

## 2. Patients and Methods

### 2.1. Patients

Primary breast cancer patients (*n* = 5153) with invasive Stage I-III cancer who underwent curative surgery and who had data for ER, PgR, Ki-67 and HER2 status from January 2002 to March 2021 at Kumamoto City Hospital and Kumamoto Shinto General Hospital were enrolled in the current study. Patients with non-invasive and Stage IV cancer were excluded from this study.

The distribution of cases according to menopausal status was investigated based on each subtype, and the clinicopathological characteristics and prognosis were compared between pre- and postmenopausal TNBC patients. Menopause was defined as the cessation of menstrual bleeding for a period of one year at the initial diagnosis of breast cancer. The study protocol was approved by the Institutional Review Board at Kumamoto Shinto General Hospital (2021-J13-006). The clinicopathological factors investigated were menopausal status, nodal status, tumor size, nuclear grade, ER/PgR and HER2 status, p53 overexpression and the Ki-67 index value. Invasive breast cancer was divided into 5 subtypes based on the immunohistochemistry (IHC) data derived from ER/PgR, HER2 and the Ki-67 index values (cutoff point: 20%). Radiotherapy was conducted on the residual breast after breast-conserving surgery (BCS), and to the chest wall or regional lymph node after mastectomy. The criteria for choosing radiotherapy were tumors that were >5 cm, cancer cells located in more than three lymph nodes, and cancer cells located in the surgical margins (i.e., the skin or muscles). Informed consent to participate in this study was obtained from the patients.

### 2.2. Histopathological Examination

The histological type was determined according to the World Health Organization (WHO) classification and the histological type of the resected specimens was re-evaluated in each of the TNBC cases (105 in the premenopausal and 328 in the postmenopausal cases).

IHC for ER, PgR, p53, Ki-67 and HER2 was conducted using the auto-stainer [10] procedure (Benchmark XT; Ventana Medical Systems, Inc., Tucson, AZ, USA). The positive cell rates for ER/PgR were determined by IHC using the monoclonal antibody (rabbit ER-antibody SP1/PgR-antibody 1E2) and a value of ≥1% was considered positive. The antibodies used for IHC were HER2 (clone 4B5), p53 (clone DO7) and Ki-67 (clone MIB-1). Determining the positive cell rate for Ki-67 was carried out by calculating the number of tumor cells in the hot spot, and a value of ≥500 cancer cells was the indicator for positive tumors. The status of p53-positive cells ≥50% was classified as p53 overexpression [11]. The HER2 status was dichotomized into positive and negative cases using IHC and the FISH test. Cases with IHC3+ or FISH amplified were categorized as HER2 positive.

### 2.3. Breast Cancer Subtypes and Adjuvant Therapy

Luminal A type was determined in cases with hormone receptor (HR) positive (ER/PgR) and HER2 negative tumors with lower Ki-67 index values (<20%), and luminal B type was determined in cases with higher Ki-67 index values (≥20%). Luminal HER2 type was categorized in cases with HR positive and HER2 positive tumors, and the HER2 enriched type in cases with HR negative and HER2 positive tumors. The TN type was categorized in cases with HR negative and HER2 negative tumors. Most of the cases with luminal type tumors underwent endocrine therapy (tamoxifen or aromatase inhibitor, LHRH agonist if younger than the age of 40) and most of the cases with TN and HER2 type received chemotherapy (anthracycline containing regimen +/− taxane, and anti-HER2 therapy if HER2 positive). The Japanese government approved the use of anti-HER2 therapy (trastuzumab) in 2008.

Neoadjuvant chemotherapy (NAC) is conducted mainly on patients with locally advanced breast cancer. NAC was performed on 59 premenopausal and 76 postmenopausal patients with TNBC. The pathologic complete response (pCR) using NAC was confirmed in cases with absence of invasive cancer cells in the breast (ypT0/is).

### 2.4. Statistical Analysis

The categorial variables were compared by using the chi-square test and the Fisher’s exact test (Table 1, Table 2, Table 3 and Table 4). DFS and overall survival (OS) rate were shown using the Kaplan-Meier estimates and were compared using the log-rank procedure. Uni- and multivariate analyses of DFS were conducted using the Cox proportional hazards regression model (SPSS version 21). The median follow-up period was 95.0 months.

TN: triple negative (breast cancer).

## 3. Results

### 3.1. Breast Cancer Subtypes and Menopausal Status

Of the 5153 patients, 64.3% were postmenopausal and 35.7% were premenopausal. There were significant differences in menopausal status among the subtypes (Table 1). TNBC was frequently observed in postmenopausal patients, and Luminal B and Luminal/HER2 subtypes were more common in premenopausal patients (*p* < 0.0001).

**Table 1:** Breast Cancer Subtypes and Menopausal Status.

Subtype	Menopausal Status		Total	*p* Value
	Premenopause	Postmenopause		(vs. TN)
Luminal A	616 (33.2)	1238 (66.8)	1854	<0.0001
Luminal B	778 (41.6)	1090 (58.4)	1868	<0.0001
Luminal/HER2	159 (42.2)	218 (57.8)	377	0.046
HER2 enriched	124 (30.9)	277 (69.1)	401	<0.0001
Triple Negative	165 (25.3)	488 (74.7)	653	-
Total	1842 (35.7)	3313 (64.3)	5153	*p* <0.0001

TN: triple negative (breast cancer).

### 3.2. DFS According to Menopausal Status in Each Subtype and OS in TNBC

**Figure 1:**
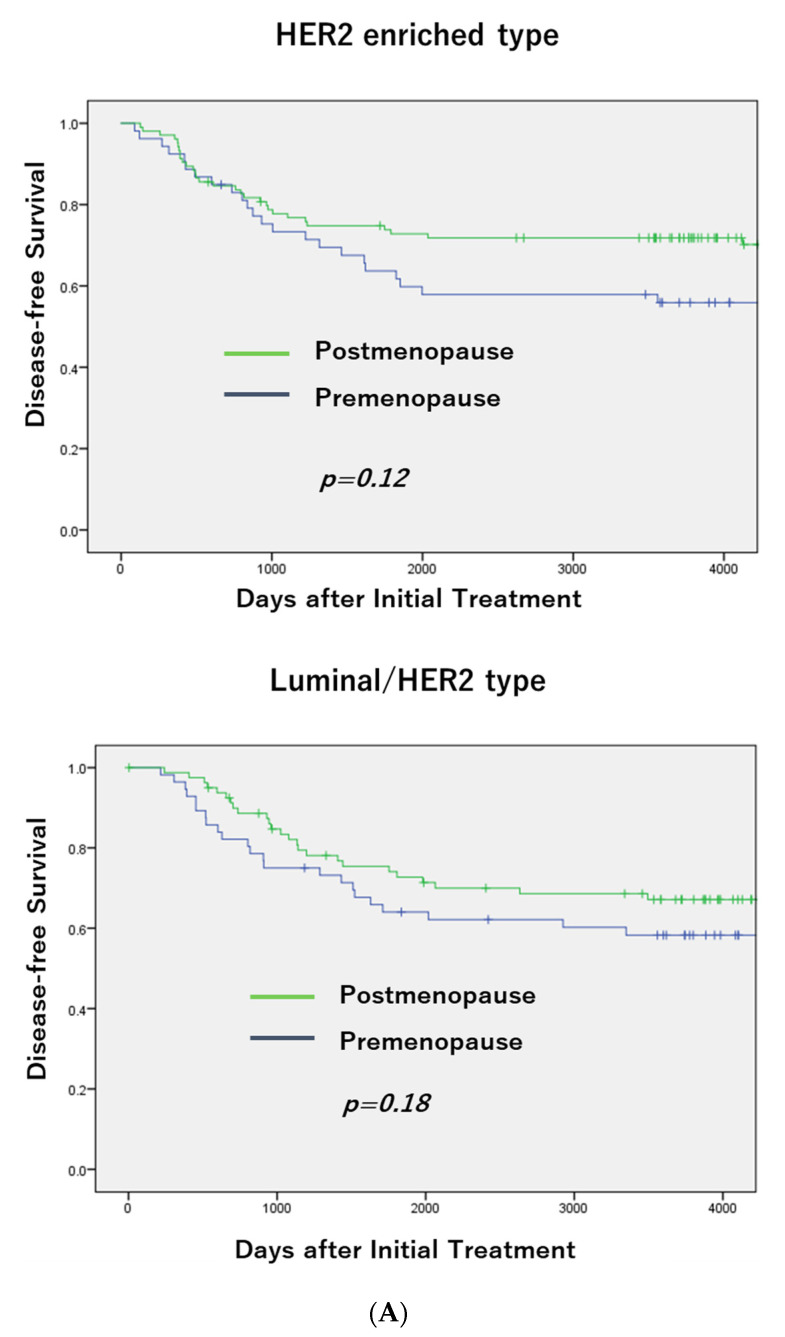

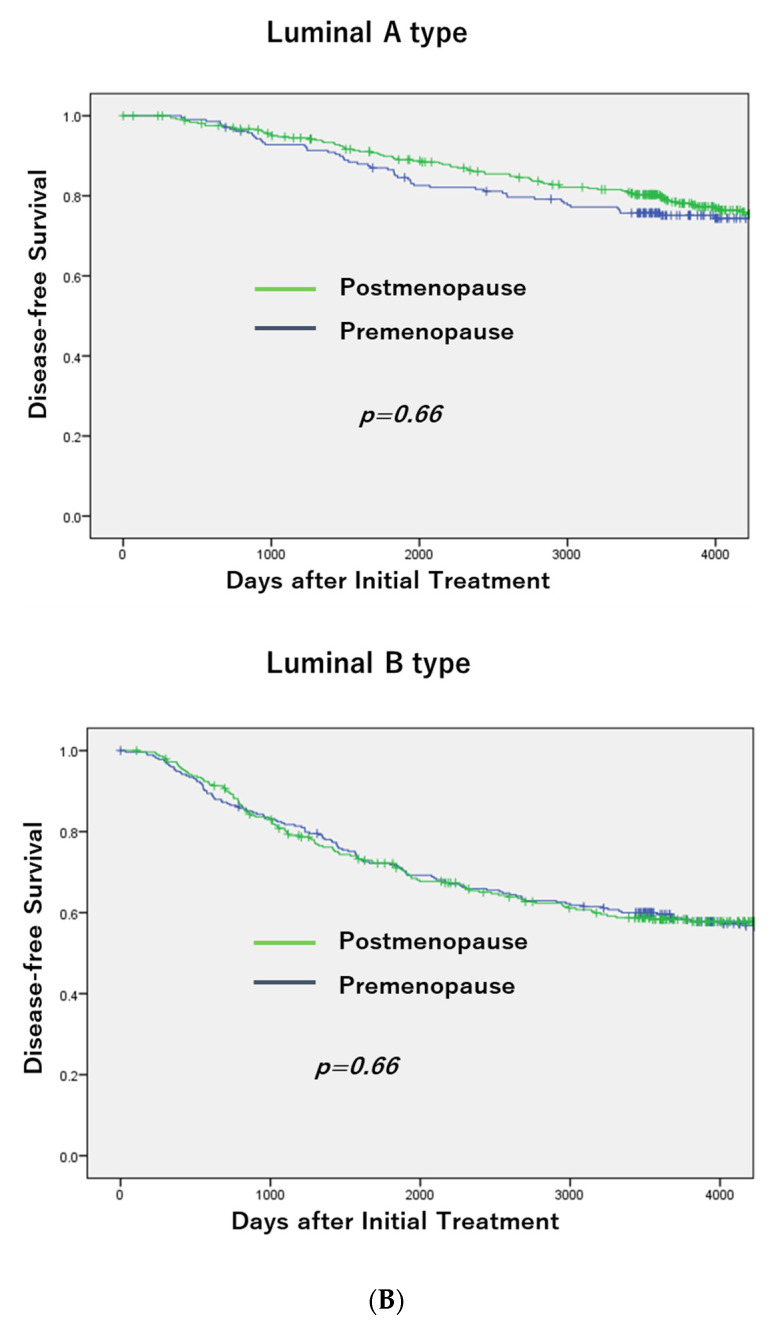

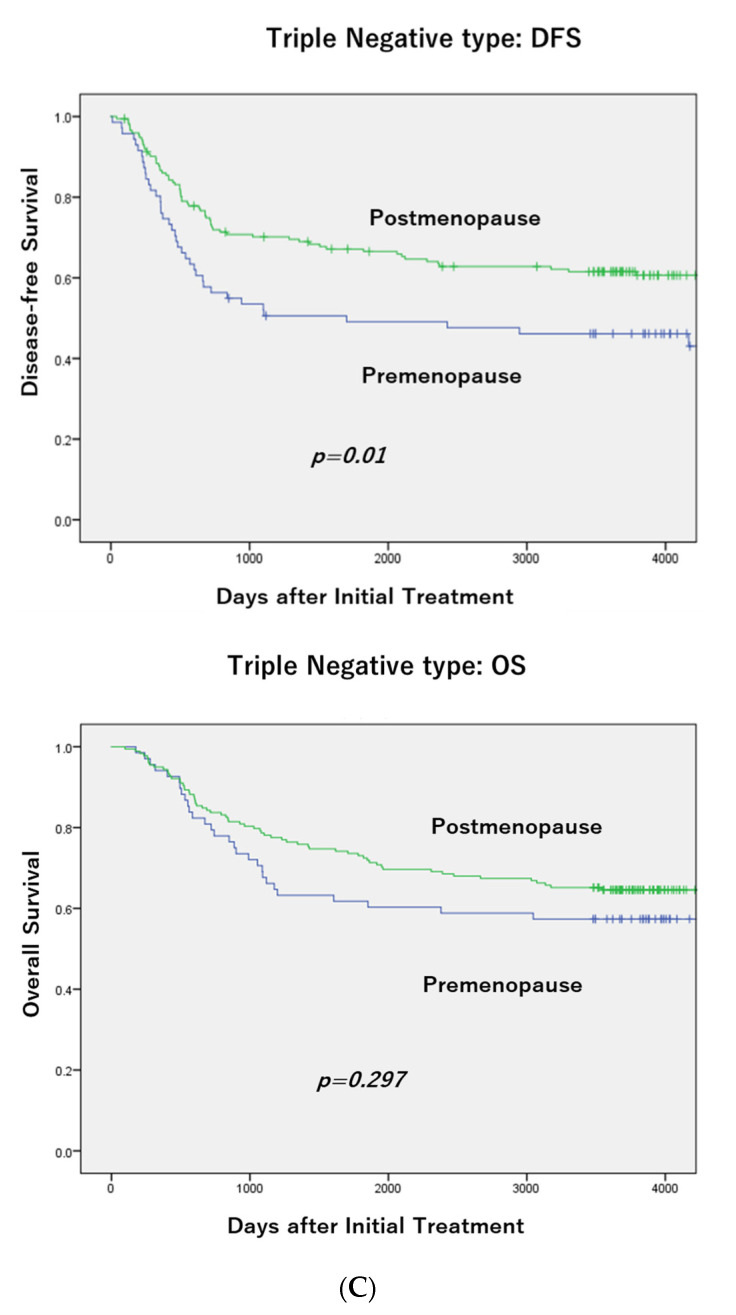
DFS according to Menopausal Status in Each Subtype and OS in TNBC. There was no difference in DFS between pre- and postmenopausal patients with the HER2 positive and Luminal A/B subtypes (**A**,**B**), but a significant difference (*p* = 0.01) was seen in patients with TNBC (premenopausal patients had a poorer DFS than postmenopausal patients; (**C**)). Moreover, postmenopausal patients had a more favorable OS than premenopausal patients, but the difference was not significant.

There was no difference in DFS between pre- and postmenopausal patients with the HER2 positive and Luminal A/B subtypes (Figure 1A,B), but a significant difference (*p* = 0.01) was seen in patients with TNBC (premenopausal patients had a poorer DFS than postmenopausal patients; Figure 1C). Moreover, postmenopausal patients had a more favorable OS than premenopausal patients, but the difference was not significant.

### 3.3. Clinicopathological Characteristics and Menopausal Status in TNBC

Menopausal status did not correlate with tumor size and nodal status in the TNBC cases (Table 2). On the other hand, premenopausal patients significantly correlated with a higher Ki-67 index value (*p* < 0.0001), p53 overexpression (*p* < 0.0001) and a higher nuclear grade (*p* = 0.045). The Ki-67 index was divided into two separate groups (cut-off point: 50%) before analyzing the factors for DFS in TNBC. Moreover, the BCS rate in premenopausal patients was significantly higher than in postmenopausal patients.

**Table 2:** Clinicopathological Characteristics and Menopausal Status in TNBC.

Variables	Menopausal Status		Total	*p* Value
	Premenopause	Postmenopause		
Tumor Size				
≤2cm	89 (23.2)	294 (76.8)	383	
>2cm	76 (28.1)	194 (71.9)	270	0.17
Nodal Status				
n0	113 (24.8)	342 (75.2)	455	
n+	52 (26.4)	145 (73.6)	197	0.7
Ki-67				
≤20%	6 (8.1)	68 (91.9)	74	
21–49%	26 (15.4)	143 (84.6)	169	<0.0001
≥50%	133 (32.6)	275 (67.4)	408	
p53 Overexpression				
without	61 (17.8)	282 (82.2)	343	
with	93 (32.5)	193 (67.5)	286	<0.0001
Nuclear Grade				
1	16 (16.3)	82 (83.7)	98	
2	31 (22.8)	105 (77.2)	136	0.045
3	116 (28.0)	299 (72.0)	415	
Surgical Procedure				
Total Mastectomy	52 (16.9)	255 (83.1)	307	
Breast Conserving Surgery	110 (32.8)	227 (67.4)	337	<0.0001

### 3.4. Histological Type and Menopausal Status in TNBC

The number of cases with invasive breast carcinoma of no special type was smaller in the postmenopausal group than in the premenopausal group (*p* = 0.029) in the TNBC cases (Table 3), and there were some special histological types in the postmenopausal group (i.e., invasive lobular carcinoma, metaplastic carcinoma, mucinous carcinoma, and adenoid cystic carcinoma).

**Table 3:** Histological Type and Menopausal Status in TNBC.

Histological Type	Premenopause	Postmenopause	*p* Value
IBC, NST	95 (90.5) *	267 (81.4) *	* 0.029
(with squamous differentiation)	(2)	(5)	
Ca w/APO	6 (5.7)	32 (9.8)	
Invasive lobular carcinoma	1 (1)	15 (4.6)	
Metaplastic carcinoma		8 (2.4)	
Mucinous carcinoma		1 (0.3)	
Adenoid cystic carcinoma		2 (0.6)	
Others	3 (2.9)	3 (0.9)	
Total	105	328	

IBC, NST: Invasive breast carcinoma of no special type. Ca w/APO: Carcinoma with apocrine differentiation. * IBC vs. others.

### 3.5. Pre- and Post-Operative Adjuvant Chemotherapy and Menopausal Status in TNBC

Premenopausal patients often received (neo-) adjuvant chemotherapy (*p* < 0.0001), but there was no significant difference in the pCR rate between the pre- and postmenopausal status (Table 4).

**Table 4:** Pre- and Post-Operative Adjuvant Chemotherapy and Menopausal Status in TNBC.

Variables	Menopausal Status		Total	*p* Value
	Premenopause	Postmenopause		
Adjuvant Chemotherapy				
without	10 (6.3)	149 (93.7)	159	
with	155 (31.4)	339 (68.6)	494	<0.0001
Neoadjuvant Chemotherapy				
without	106 (20.5)	412 (79.5)	518	
with	59 (43.7)	76 (56.3)	135	<0.0001
Effect of NAC				
pCR	27 (42.2)	37 (57.8)	64	
non-pCR	32 (45.1)	39 (54.9)	71	0.75

NAC: Neo-adjuvant chemotherapy, pCR: pathological complete response.

### 3.6. Uni- and Multivariate Analysis of Factors for DFS in TNBC

**Figure 2:**
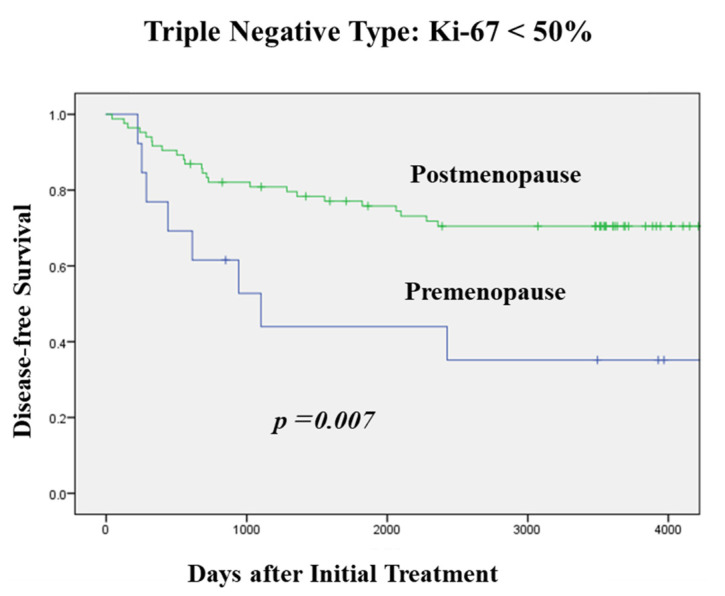

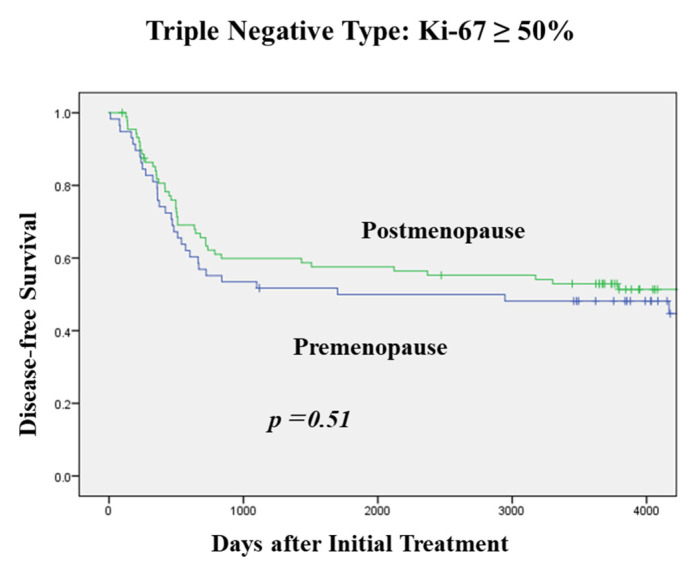
DFS according to Menopausal Status and Ki-67 Index Value in TNBC. There was no difference in DFS between the menopausal status in cases with a high Ki-67 index value (≥50%). On the other hand, in the cases with a low Ki-67 index value (<50%), the postmenopausal patients had a significantly higher DFS rate.

Figure 2 shows the DFS according to menopausal status and the Ki-67 index value in cases with TNBC. Moreover, there was no difference in DFS between the menopausal status in cases with a high Ki-67 index value (≥50%). On the other hand, in cases with a low Ki-67 index value (<50%), the postmenopausal patients had a significantly higher DFS rate.

A univariate analysis revealed that tumor size, nodal status, Ki-67, nuclear grade and menopausal status were significant factors for DFS (Table 5). Moreover, a multivariate analysis revealed that menopausal status, nodal status and tumor size were significant factors for DFS in TNBC.

## 4. Discussion

Menopausal status significantly correlated with breast cancer subtypes. TNBC was frequently seen in postmenopausal patients, and Luminal B and Luminal/HER2 subtypes were more common in premenopausal patients. There was no difference in DFS between the pre- and postmenopausal patients in the Luminal A/B and HER2 subtypes, but a significant difference was seen in patients with TNBC (premenopausal patients had worse DFS than postmenopausal patients).

In terms of the clinicopathological characteristics and menopausal status in cases with TNBC, menopausal status did not significantly correlate with tumor size and nodal status. However, the premenopausal patients had a significantly higher Ki-67 index value, p53 overexpression, and a higher nuclear grade. A pattern of natural germline alterations in TNBC may also be different from that found in the other subtypes [12]. Some of the TNBC cases (≤15%) have germline mutations in the BRCA1 and BRCA2 genes. Moreover, the TNBC subtype is frequently seen in breast tumors arising in the BRCA1 mutation carriers (70%) and BRCA2 carriers (16–23%) [12]. Lehmann et al. [13] analyzed gene expression profiles of 587 TNBC cases and recognized 6 TNBC subtypes, including 2 basal-like (BL1 and BL2), an immunomodulatory (IM), a mesenchymal (M), a mesenchymal stem-like (MSL), and a luminal androgen receptor (LAR) subtype. The BL1 and BL2 subtypes responded to cisplatin, the M and MSL subtypes responded to the inhibition of the phosphoinositide 3-kinase (PI3K)/mTOR and Abl/Src pathways, and the LAR subtype was sensitive to the AR antagonist, suggesting that gene expression analysis could help identify appropriate targeted therapies for patients with TNBC [14]. It is therefore important to understand the diversity of the TNBC subtypes.

Premenopausal cases were more often likely to undergo BCS even in cases with larger tumors using neoadjuvant chemotherapy, but the margin positive rate was not high. Our previous study found that the margin positive rate was 5.9% in cases with BCS (within 5 mm from the edge of the margin) [15]. However, there were only a few cases where the tumor cells were found at the excised margin.

Based on the histological findings, the majority of TNBC cases are of ductal origin; however, several other phenotypes include metaplastic, apocrine and adenoid cystic carcinoma [16]. Cases with triple negative apocrine cancer (TNAC) had a more favorable outcome than cases with TNBC, and chemotherapy might have contributed to the survival advantages in TNAC patients. Moreover, TNAC patients tended to be older at diagnosis than TNBC patients [17]. Lehmann et al. also reported that IHC showed higher levels of AR expression in TNBC of the LAR subtype [13]. Gerratana et al. found that the LAR subtype was associated with a more favorable prognosis, chemotherapy unresponsiveness and a lower pCR rate after NAC [18]. Wang et al. [19] conducted a meta-analysis of 2826 TNBC cases in 13 trials from 2007 to 2015, and found that 24.4% of the TNBC cases expressed AR, and these cases significantly correlated with postmenopausal status, low tumor grade, and a high risk of nodal involvement.

The PgR positive rate in the postmenopausal cases was lower than the PgR positive rate in the premenopausal cases [20]. Patients with PgR negative tumors were older than those with PgR positive tumors [21]. ER does not function sufficiently in some PgR negative cases and the tumor is therefore no longer dependent on estrogen for growth and survival. Some studies have also found that there is a positive correlation between the PgR status and low estrogen levels in postmenopausal cases [22,23]. Thus, postmenopausal cases tend to have more PgR negative tumors. Moreover, patients with low PgR and low Ki-67 index values had a better prognosis [24]. The DFS in postmenopausal TNBC cases was closely associated with a Ki-67 index value; a more favorable DFS was seen in TNBC cases with a lower Ki-67 index value.

There are three potential limitations in this study. First, the data were derived from a retrospective analysis, but there is a sufficient observation period (95.0 months) in more than 5000 cases. Second, the subtypes were determined by biomarkers derived from IHC. However, IHC is inexpensive and does not require the use of expensive technology. Third, menopausal status was determined at the initial diagnosis. Amenorrhea due to treatment was not taken into account in this study. Moreover, this study focused on TNBC, which might be unresponsive to endocrine manipulation.

In conclusion, menopausal status significantly correlates with breast cancer subtypes. TNBC was often seen in postmenopausal patients and these patients tend to have more favorable factors (low Ki-67, absence of p53 overexpression, and a low nuclear grade) and a better DFS than premenopausal patients. These findings suggest that menopausal status is an important factor in evaluating the biology and prognosis in TNBC cases.

## Figures and Tables

**Table 5:** Uni- and Multivariate Analysis of Factors for DFS in Triple Negative Breast Cancer.

Variable	Category	Univariate Analysis		Multivariate Analysis	
		HR (95% CI)	*p* Value	HR (95% CI)	*p* Value
Tumor Size	≤2 cm vs. >2 cm	1.93 (1.30–2.86)	0.001	1.58 (1.05–2.37)	0.027
Nodal Status	N− vs. n+	4.14 (2.77–6.18)	<0.0001	3.91 (2.59–5.90)	<0.0001
Ki-67	≤20% vs. >20%	2.78 (1.13–6.83)	0.026	1.89 (0.73–4.87)	0.19
p53 overexpression	without vs. with	1.16 (0.79–1.72)	0.43	-	-
Nuclear Grade	1, 2 vs. 3	1.68 (1.12–2.51)	0.011	1.42 (0.93–2.17)	0.1
Menopausal Status	Pre- vs Post-	0.61 (0.41–0.91)	0.014	0.54 (0.36–0.82)	0.003

HR: hazard ratio. CI: confidence interval.

## Data Availability

The datasets used and/or analyzed during the current study are available from the corresponding author on reasonable request.
